# Soil microbial community and influencing factors of different vegetation restoration types in a typical agricultural pastoral ecotone

**DOI:** 10.3389/fmicb.2024.1514234

**Published:** 2025-01-23

**Authors:** Pei Huang, Hanyu Shi, Lina Jiang, Duoping Zhu, Zefeng Zhou, Zhenhong Hou, Xingyu Ma

**Affiliations:** ^1^College of Life Science, Capital Normal University, Beijing, China; ^2^Institute of Ecological Conservation and Restoration, Chinese Academy of Forestry, Beijing, China; ^3^State Forestry Administration Dunhuang Desert Ecosystem Location Research Station, Dunhuang, China; ^4^Institute of Forest Resource Information Techniques, Chinese Academy of Forestry, Beijing, China; ^5^Graduate Department, Chinese Academy of Forestry, Beijing, China

**Keywords:** agricultural pastoral ecotone, afforestation restoration, co-occurrence network analysis, microbial diversity, network complexity

## Abstract

Microbial network complexity is an important indicator for assessing the effectiveness of vegetation restoration. However, the response of the microbial network complexity of bacteria and fungi to different vegetation restoration types is unclear. Therefore, in this study, we selected four vegetation restoration types (*Pinus sylvestris* var. *mongholica*, *Larix principis- rupprechtii*, *Populus tomentosa*, and *Ulmus pumila*), while selected the nature grassland as a control, in the Zhangjiakou Tunken Forest Farm, which is a typical agricultural pastoral ecotone in northern China, to investigate the response of soil microbial diversity and network complexity to different vegetation restoration types. Our result showed that the bacterial Shannon and Chao indices of *P. sylvestris* var. *mongholica* were significantly 7.77 and 22.39% higher than those of grassland in the 20–40 cm soil layer, respectively. The fungal Chao indices of *U. pumila* were significantly 85.70 and 146.86% higher than those of grassland in the 20–40 cm and 40–60 cm soil layer, respectively. Compared to natural grassland, soil microbial networks became more complex in plantation forests restoration types (*P. sylvestris* var. *mongholica*, *L. principis- rupprechtii*, *P. tomentosa*, and *U. pumila*). Microbial network complexity increased with soil carbon and nitrogen. *P. tomentosa* is suitable for planting in the agricultural pastoral ecotone of Zhangjiakou, because of its high soil carbon, nitrogen and microbial network complexity. Bacterial community composition was found to be closely related to soil organic carbon (SOC), total nitrogen (TN), while that of fungi was closely related to SOC, clay and silt content. This improvement in microbial complexity enhances the ecological service function of the agricultural pastoral ecotone. These findings offer theoretical basis and technical support for the vegetation restoration of ecologically fragile areas in agricultural pastoral ecotone.

## 1 Introduction

Desertification is a major global ecological and environmental problem caused by natural factors (climate change) and human activities (overgrazing and overcultivation), and it results in land degradation in drylands (Yao et al., 2023). Vegetation restoration is an important strategy to manage land desertification and achieve habitat restoration in drylands (Zhang et al., 2022). Vegetation restoration enhances ecosystem nutrient cycling and improves soil quality through plant–soil interactions, thereby contributing to effective desertification management. Different vegetation restoration patterns significantly affect the diversity and productivity of vegetation, which in turn affect soil carbon sequestration potential and microbial characteristics (Xu et al., 2011). As the key components of terrestrial ecosystems, soil microorganisms play an important role in regulating ecological processes, including nutrient cycling, pathogen control, and plant productivity (Pedrinho et al., 2024). The increase of microbial diversity develops stable and resilient soil ecosystems, while the decrease of microbial diversity is detrimental to ecosystem sustainability (Wang et al., 2024). There is thus increasing emphasis in studying soil microbial diversity in different vegetation restoration types for ecological restoration in drylands.

China’s agricultural pastoral ecotone is a dryland connecting the eastern farming area and the western grassland pastoral area, which has a fragile ecosystem and serious desertification problems (Li et al., 2022). Usually, vegetation restoration in the agricultural pastoral ecotone are mostly grassland-based, accompanied by natural and plantation forests and shrubs (Yang et al., 2014). Currently, studies in agricultural pastoral ecotone have focused on grasslands, farmlands and scrub (Cui et al., 2019; Felipe-Lucia et al., 2020), while few studies have investigated plantation forest, particularly in terms of soil microbial communities. This limits the assessment of the ecological efficacy of these vegetation restoration types from the microbial point of view. It is generally believed that microbial activity is the highest in the top soil layer (0–20 cm), but microorganisms in deeper soil layers still play important roles in nutrient metabolism, energy cycling and soil formation (Jiao et al., 2018). Therefore, the study of microbial diversity in multilayer soils can help to understand the important role of soil microorganisms in the ecological restoration of agricultural pastoral ecotone.

Microbial network analysis is an effective way to understand the structure of soil microbial networks, and it has been widely used by microbial ecologists (Chen et al., 2022; Dong et al., 2022). Different species or taxa are linked together by exchanging matter, energy, and information, which results in complex interactions such as competition and mutually beneficial symbiosis (Wang et al., 2023). These complex interconnections among microorganisms can be represented as co-occurring networks, where microbial taxa act as nodes and their relationships as links (Liu et al., 2023; Zhang et al., 2023). Furthermore, microbial diversity and interactions among taxa may vary over time, space, or environment (Xu et al., 2022). It has been shown that soil microbial diversity positively influences ecosystem functioning indirectly mainly by promoting network complexity (Zhai et al., 2024). Understanding microbial network complexity is essential for assessing the level of functional recovery in degraded restored ecosystems. However, the effect of different vegetation restoration types on microbial network complexity in the agricultural pastoral ecotone is unknown.

Zhangjiakou is located in northwestern Hebei and belongs to a typical agricultural pastoral ecotone. It is a crucial water source conservation area and ecological security barrier for the capital region of China (Zhou et al., 2021). Additionally, this area is a major hub for production and living activities, where frequent human activities render the ecological environment sensitive and fragile. It is also an important ecological corridor in the upper reaches of the Beijing–Tianjin–Hebei Urban Agglomeration, which plays an extremely important ecological service function (Li et al., 2020). Based on the important ecological service function of Zhangjiakou area and its status as a key ecological conservation area in the upper reaches of Beijing–Tianjin–Hebei, in this study, we selected four vegetation types [*Pinus sylvestris* var. *mongholica* plantation (ZS), *Larix principis- rupprechtii* plantation (LS), *Populus tomentosa* plantation (PS) and *Ulmus pumila* plantation (YS)] with the same site conditions and similar restoration years in Zhangjiakou Tunken Forest Farm, while selected natural grassland (GL) as a control. This study aimed to investigate the effects of various vegetation restoration types on the diversity of soil microbial communities and the complexity of microbial networks in a typical agricultural pastoral ecotone in Zhangjiakou. We hypothesized that (1) different vegetation restoration types may alter soil nutrient status, with broadleaf forests (PS, YS) having higher nutrient content than coniferous forests (ZS, LS), and coniferous forests having higher nutrient content than natural grassland (GL); (2) changes in soil nutrients content are the key drivers of bacterial and fungal community characteristics under different vegetation restoration types; (3) different vegetation restoration types changes microbial network complexity.

## 2 Materials and methods

### 2.1 Experimental site

The study area is located in Tunken Forest, Kangbao County, Zhangjiakou City, Hebei Province, China (114.47°E, 41.54°N), which belongs to a typical agricultural pastoral ecotone. The area has a semi-arid monsoon climate in the north temperate zone. It has high temperature and little rain in summer and is cold and dry in winter. The average annual temperature is 2.1°C, and a frost-free period lasts for 90–105 days. The average annual precipitation is about 300 mm, with rain and heat concentrated in the same season, primarily from July to September. The main afforestation species are ulmus, populus, pinus, and larch. The regional soil is chestnut soil and meadow soil, with medium soil fertility.

### 2.2 Sample collection and pretreatment

In May 2023, natural grassland (GL), larch plantation forest (LS), pinus plantation forest (ZS), populus plantation forest (PS), and ulmus plantation forest (YS) with similar elevation, flat terrain, and basically the same restoration age were selected within the Tunken Forest Farm for the study. The selected sites all had similar soil characteristics prior to afforestation. The plantation forests had fewer understory plants and lower surface biomass than natural grassland (Table 1). Three sample plots (1 m × 1 m) were randomly selected in each sample site. The five-point sampling method was used to collect soil samples of 0–20 cm, 20–40 cm, and 40–60 cm using a soil auger with an inner diameter of 5 cm around the sample points and at the center. Visible matter, such as plant stubs, stones and gravels in the samples, were removed. Then soil samples from the same soil layer were immediately mixed to form a single soil sample. The mixed soil samples were divided into two parts, which were placed into sealed bags and sterilized tubes for microbiological sampling. The soil sample in the sealed bag was air-dried and used to determine soil physical and chemical properties; the soil sample in the sterilized tubes for microbiological sampling was returned to the laboratory with an ice pack and stored at −80°C for high-throughput sequencing analysis.

**Table 1:** Basic information of the sample site.

Vegetation type	Altitude/m	Restoration time/a	Line spacing/m	Plant spacing/m	Average height/m	Average diameter at breast height/cm	Average ground diameter/cm	Main herbs	Forest understory surface biomass/g/m^2^
Nature grassland	1,440	45	–	–	–	–	–	*Setaria viridis*, *Eragrostis Pilosa, Chloris virgata*, *Taraxacum mongolicum*	216.62 ± 10.24
Larch	1,435	49	6	2	12.1 ± 0.5	14.4 ± 2.6	18.2 ± 3.0	*Plantago asiatica*, *Setaria viridis*	107.15 ± 7.62
Pinus	1,438	42	6	3	14.1 ± 1.3	25.1 ± 3.9	30.6 ± 4.2	*Setaria viridis*, *Chloris virgata*	87.42 ± 6.56
Populus	1,446	48	6	3–6	12.9 ± 0.8	25.6 ± 5.0	30.5 ± 7.3	*Cleistogenes hancei*, *Carex bohemica*	91.88 ± 8.79
Ulmus	1,440	50	3	1–2	10.2 ± 1.2	16.1 ± 1.4	21.6 ± 3.1	*Agrimonia pilosa*, *Carex bohemica*	113.44 ± 4.92

Mean ± standard deviation, *n* = 10. Forest understory surface biomass is the above-ground biomass of understory shrubs and grasses.

### 2.3 Soil property measurements

Soil pH was measured by a pH meter (a 1:2.5 soil/water mixture), soil organic carbon (SOC) was measured by the potassium dichromate volumetric method, total carbon (TC) was measured by the high-temperature scorching method, total nitrogen (TN) was measured by the semi-micro Kjeldahl method (Bao, 2000), and soil particle size was measured using a Mastersizer 3000 laser particle sizer. Soil particle size grading was based on the American system of soil grain size: clay (<2 μm), silt (2–50 μm), and sand (50–2000 μm) (Chen et al., 2020).

### 2.4 Soil DNA extraction and high-throughput sequencing

Total DNA extraction from 0.5 g soil samples was performed according to the FastDNA^®^ Spin Kit for Soil (MP Biomedicals, United States) instructions, and DNA extraction quality was checked using 1% agarose gel electrophoresis. DNA concentration and purity were determined using a NanoDrop2000.

PCR was performed on a GeneAmp 9700 PCR system. Primers 338F (5′-ACTCCTACGGGAGGCAGCAG-3′) -806R (5′-GGACTACHVGGGTWTCTAAT-3′) and ITS1F (5′-CTTGGTCATTTAGAGGAAGTAA-3′) -ITS2R (5′-GCTGCGTTCTTCATCGATGC-3′) were used for soil bacterial 16S rRNA genes in the V3-V4 hypervariable regions and soil fungal internal transcribed spacer (ITS) region, respectively (Sun et al., 2017). Amplification products were detected by 2% agarose gel electrophoresis and recovered from the gel using the AxyPrep DNA Gel Extraction Kit, washed with Tris–HCl and verified by 2% agarose gel electrophoresis (Hu et al., 2017). PCR products were quantified using a QuantiFluorTM-ST fluorometer, and samples were adjusted according to sequencing needs.

Sequencing was performed on the Illumina MiSeq platform by Shanghai Major Biomedical Technology Company. The high-throughput sequencing data were deposited in the NCBI Sequence Read Archive (BioProject ID PRJNA1015983, study accession number SRP460000).

### 2.5 Sequence analysis

Sequence analysis was performed using Uparse software (v7.0.1090) to cluster valid data from soil samples. Sequences shorter than 200 bp, ambiguous bases, and sequences with an average mass less than 25 were removed (Hu et al., 2017). Chimeric sequences were removed using USEARCH (v11). Unique sequences >97% similarity levels were clustered into an operational taxonomic units (OTU) (Nguyen et al., 2016). Bacterial and fungal sequences were classified by comparing the representative sequences of OTUs with Silva version 138 16S rRNA and UNITE version 8.0 databases, respectively (Zhou et al., 2020). Diversity indices Chao and Shannon were calculated using the Qiime (v1.9.1) platform.

### 2.6 Statistical analysis

Soil physicochemical properties and microbial diversity were analyzed and plotted using R (4.2.1). We used microbial OTU richness as the metrics of, and calculated, the microbial *α*-diversity. Soil physicochemical properties and microbial α-diversity were compared by one-way ANOVA, and significance of differences was compared by the LSD method. The data of soil physicochemical properties and microbial α-diversity are presented as mean values ± standard deviation (SD). Beta diversity of soil microbial communities was resolved by principal co-ordinates analysis (PCoA) based on the weighted unifrac distance algorithm, and differences between groups were tested using ANOSIM similarity analysis (number of permutations = 999) with the R “vegan” package. Linear regression analysis was used to assess the relationship between microbial community composition and environmental factors.

### 2.7 Microbial co-occurrence network construction

The construction of microbial co-occurrence networks used with Pearson’s correlation coefficients matrix the R “WGCNA” package (Langfelder and Horvath, 2008). Pearson’s correlation (*p* > 0.8) and significance values (*p* < 0.01) were used for microbial network building. Topological features of the overall network (e.g., nodes, edges, average degree, modularity, density, average path length and average clustering coefficient) were calculated using the R “igraph” package (Wang et al., 2023). To obtain the microbial network complexity index, we calculated the Z-scores for some topological features (nodes, edges, average degree, average clustering coefficient, density and the reciprocal of average path length) of the microbial network individually. The Z-scores for all measured variables were averaged to obtain the complexity index of the microbial network for each locality (Wang et al., 2023). The co-occurrence networks were plotted using Gephi (0.10.1) software and visualized by the Frucherman Reingold algorithm.

## 3 Results

### 3.1 Soil physicochemical properties

Significant differences were observed in the physicochemical properties (Table 2). Soil pH was slightly alkaline and varied from 8.29 to 8.57. The soil was dominated by sand, followed by silt, and the content of clay particles was the lowest. In 0–20, 20–40, and 40–60 cm layers, total carbon (TC) in PS was significantly higher than that in GL (*p* < 0.05), with increases of 90.23, 86.73, and 109.01%, respectively. In 40–60 cm layer, the TC in YS was significantly higher than that in GL (*p* < 0.05), with an increase of 114.15%. In 0–20 cm layer, total nitrogen in PS and YS was significantly higher than that in GL (*p* < 0.05), with increases of 41.54 and 60.00%, respectively. In 20–40 cm layer, TN in PS and YS was significantly higher than that in GL (*p* < 0.05), with increases of 101.35 and 120.27%, respectively. In 40–60 cm layer, TN in the PS and ZS was significantly higher than that in GL (*p* < 0.05), with increases of 292.31 and 315.38%, respectively. In 0–20, 20–40, and 40–60 cm layers, soil organic carbon (SOC) in PS was significantly higher than that in GL (*p* < 0.05), with increases of 53.95, 82.61, and 360.00%, respectively. In 0–20 and 20–40 cm layers, SOC in YS was significantly higher than that in GL (*p* < 0.05), with increases of 60.90 and 107.41%, respectively. Therefore, compared with GL, plantation forest increased soil carbon and nitrogen, especially in PS.

**Table 2:** Physicochemical properties of the soil of the five vegetation restoration types.

Soil index	GL			LS			PS			YS			ZS		
	0–20 cm	20–40 cm	40–60 cm	0–20 cm	20–40 cm	40–60 cm	0–20 cm	20–40 cm	40–60 cm	0–20 cm	20–40 cm	40–60 cm	0–20 cm	20–40 cm	40–60 cm
pH	8.32 ± 0.09ab	8.44 ± 0.18a	8.57 ± 0.26a	8.29 ± 0.08b	8.35 ± 0.20a	8.43 ± 0.14a	8.33 ± 0.01ab	8.42 ± 0.03a	8.53 ± 0.08a	8.32 ± 0.08ab	8.37 ± 0.04a	8.44 ± 0.13a	8.44 ± 0.04a	8.40 ± 0.10a	8.40 ± 0.34a
TC (g·kg^−1^)	13.49 ± 0.89b	13.79 ± 5.28b	10.88 ± 7.62b	15.40 ± 6.74b	17.70 ± 8.98ab	12.80 ± 4.15b	25.67 ± 6.01a	25.75 ± 4.65a	22.74 ± 2.83a	21.94 ± 5.85ab	23.62 ± 7.61ab	23.30 ± 4.39a	21.19 ± 5.97ab	19.79 ± 4.31ab	17.95 ± 0.92ab
TN (g·kg^−1^)	1.30 ± 0.12c	0.74 ± 0.14bc	0.26 ± 0.12b	1.08 ± 0.40c	0.53 ± 0.45c	0.63 ± 0.21ab	1.84 ± 0.28ab	1.49 ± 0.36a	1.02 ± 0.27a	2.08 ± 0.39a	1.63 ± 0.08a	0.90 ± 0.12ab	1.46 ± 0.11bc	1.24 ± 0.45ab	1.08 ± 0.71a
SOC (g·kg^−1^)	11.51 ± 0.81c	7.42 ± 1.17b	3.75 ± 0.84b	10.72 ± 3.31c	8.30 ± 1.00b	9.50 ± 4.13ab	17.72 ± 3.43ab	13.55 ± 3.23a	17.25 ± 6.78a	18.52 ± 2.85a	15.39 ± 0.84a	8.93 ± 0.39b	13.25 ± 1.67bc	11.03 ± 4.64ab	10.17 ± 5.96ab
Clay content (%)	5.60 ± 0.84c	5.07 ± 1.72a	4.53 ± 1.21a	7.62 ± 0.81ab	10.22 ± 5.68a	6.37 ± 1.77a	9.12 ± 0.79a	7.60 ± 0.52a	10.20 ± 1.17a	3.67 ± 1.53d	5.48 ± 2.50a	4.28 ± 0.61a	6.49 ± 0.15bc	7.09 ± 3.10a	11.99 ± 11.43a
Silt content (%)	31.24 ± 2.59b	26.17 ± 7.36a	29.14 ± 1.79c	34.57 ± 4.60ab	39.05 ± 6.82a	34.31 ± 2.99bc	41.34 ± 2.19a	40.38 ± 3.02a	46.00 ± 2.79a	31.75 ± 3.02b	36.77 ± 9.54a	34.60 ± 1.07bc	39.31 ± 6.62a	37.80 ± 13.87a	36.11 ± 6.53b
Sand content (%)	63.15 ± 3.32a	68.74 ± 9.08a	66.36 ± 1.82a	57.83 ± 5.26ab	50.73 ± 10.28a	59.33 ± 4.47a	49.55 ± 1.45c	52.01 ± 3.33a	43.78 ± 3.40a	64.58 ± 2.04a	57.73 ± 8.44a	61.11 ± 0.48a	54.19 ± 6.61bc	55.10 ± 16.23a	51.88 ± 17.31a

Mean ± standard deviation, *n* = 3. Different lowercase letters indicate significant differences among the different vegetation types for the same soil layer at the 0.05 level. GL, nature grassland; LS, larch; PS, populus; YS, ulmus; ZS, pinus; TC, total carbon; TN, total nitrogen; SOC, soil organic carbon.

### 3.2 Sequencing results of soil samples

A total of 2,636,522 (ranging from 34,619 to 63,468 sequences per sample) and 3,975,388 (ranging from 50,899 to 166,964 sequences per sample) quality sequences of the bacterial 16S rRNA and fungal ITS genes were obtained, respectively. In total, 19,957 bacterial OTUs and 5,957 fungal OTUs were obtained in the five vegetation restoration types.

### 3.3 Alpha diversity of soil microbial communities

**Figure 1:**
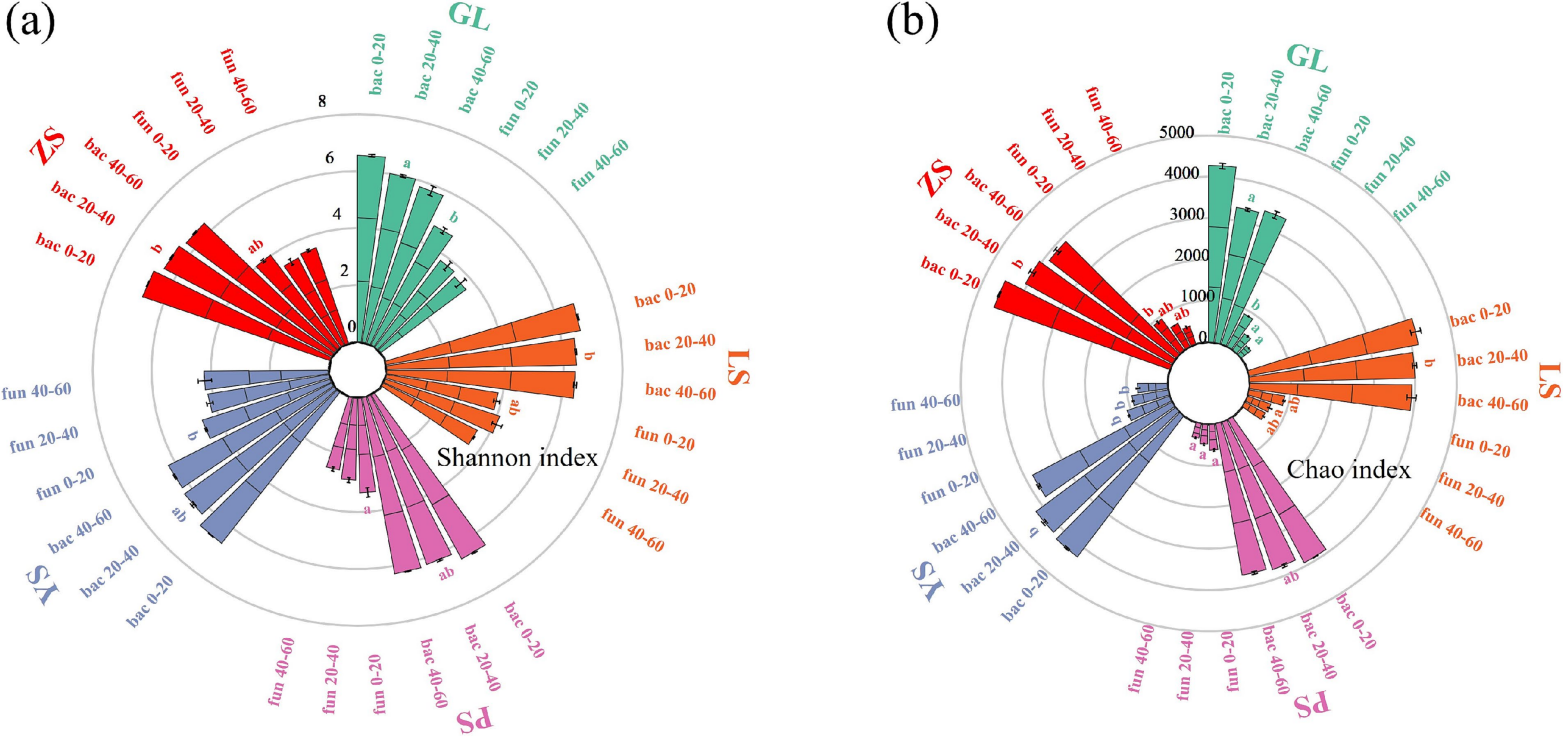
Soil microbial *α*-diversity changes in five vegetation restoration types. **(A)** Shannon index, **(B)** Chao index. GL, nature grassland; LS, larch; PS, populus; YS, ulmus; ZS, pinus. Different lowercase letters indicate significant differences among five different vegetation types for the same indicator at the 0.05 level. Bac 0–20, bac 20–40, bac 40–60 represent bacteria in the 0–20 cm, 20–40 cm, 40–60 soil layer, respectively. Fun 0–20, fun 20–40, fun 40–60 represent fungi in the 0–20 cm, 20–40 cm, 40–60 soil layer, respectively.

The bacterial Shannon of LS and ZS were significantly 7.27 and 7.77% higher than those of GL in the 20–40 cm soil layer, respectively (Figure 1). The bacterial Chao indices of LS and ZS were significantly 21.06 and 22.39% higher than those of GL in the 20–40 cm soil layer, respectively. The fungal Shannon and Chao indices of PS were significantly 29.47 and 30.17% lower than those of GL in the 0–20 cm soil layer. The fungal Chao indices of YS were significantly 85.70 and 146.86% higher than those of GL in the 20–40 cm and 40–60 cm soil layer, respectively. Overall, Shannon and Chao indices decreased with increasing depth. Generally, compared to GL, plantation forests vegetation restoration types increased microbial alpha diversity.

### 3.4 Analysis of soil microbial community composition

**Figure 2:**
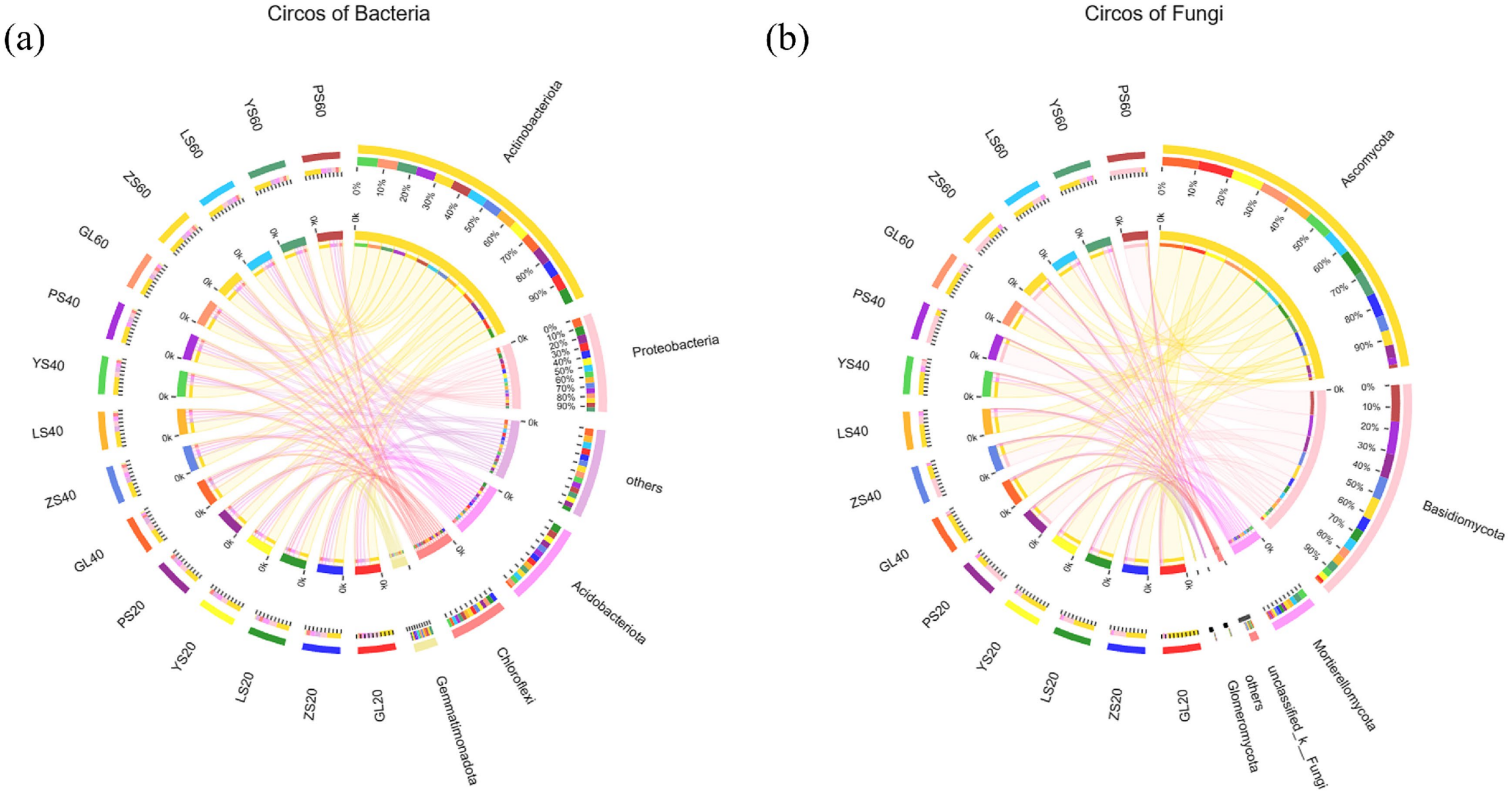
Microbial community composition changes in five different vegetation restoration types. **(A)** Bacteria, **(B)** fungi. GL20, GL40, and GL60 represent the 0–20 cm, 20–40 cm, and 40–60 cm soil layer of natural grassland soil, respectively. ZS20, ZS40, and ZS60 represent the 0–20 cm, 20–40 cm, and 40–60 cm soil layer of pinus soil, respectively. LS20, LS40, and LS60 represent the 0–20 cm, 20–40 cm, and 40–60 cm soil layer of larch soil, respectively. YS20, YS40, and YS60 represent the 0–20 cm, 20–40 cm, and 40–60 cm soil layer of ulmus soil, respectively. PS20, PS40, and PS60 represent the 0–20 cm, 20–40 cm, and 40–60 cm soil layer of populus soil, respectively.

The dominant phylum in the bacterial and fungal communities were generally consistent across the five vegetation restoration types (Figure 2). Actinobacteria, Proteobacteria, and Acidobacteriota were the primary bacterial phyla in the examined soil samples (Figure 2A), while Ascomycota and Basidiomycota were the primary fungal phyla (Figure 2B). Notably, the abundance of Ascomycota was higher in GL, LS, and YS soils with 69–88%, 59–64%, and 59–77%, respectively, while the abundance of Basidiomycota was higher in PS soils with 59–89%. The abundance of dominant phyla of fungi in the surface layer of the ZS and the lower layer of the ZS varied, with the abundance of Ascomycota in the 0–20 cm layer being high with 56%; its abundance in the 20–40 cm and 40 cm layers was also high. The abundance of Ascomycota was higher in the 0–20 cm soil layer with 56%, while the abundance of Ascomycota was higher in the 20–40 cm and 40–60 cm soil layers with 54 and 53%, respectively.

### 3.5 Beta diversity of soil microbial communities

**Figure 3:**
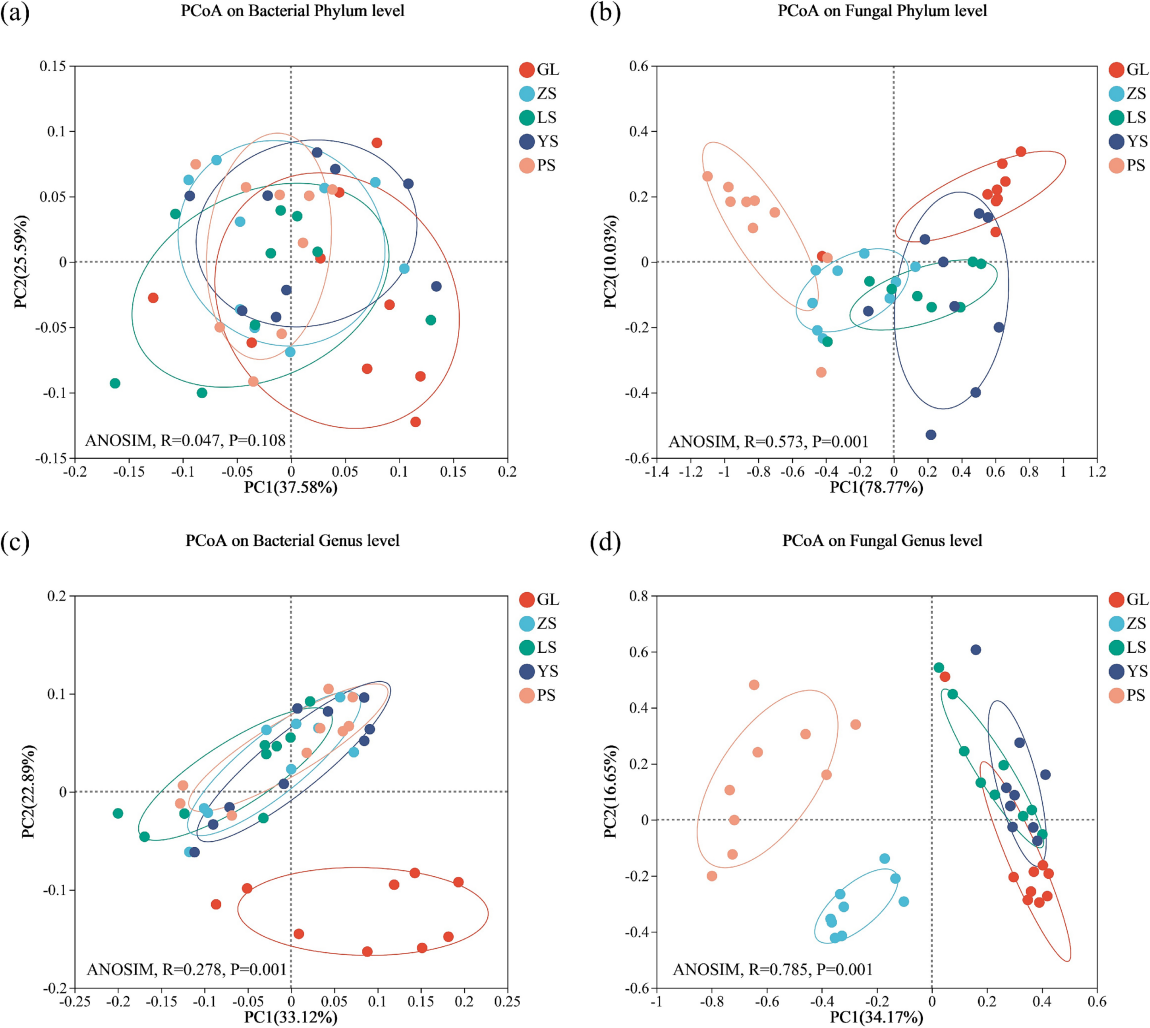
Soil microbial community *β*-diversity in five vegetation restoration types. Principal Coordinate Analysis was calculated on weighted unifrac distance. **(A)** Bacterial phylum level; **(B)** fungal phylum level; **(C)** bacterial genus level; **(D)** fungal genus level. GL, nature grassland; LS, larch; PS, populus; YS, ulmus; ZS, pinus.

Principal Coordinate Analysis was used to resolve the variability in the composition of soil bacterial and fungal communities in the five vegetation restoration types (Figure 3). The results demonstrated that, at the phylum level, the first two axes (PC1 and PC2) explained 63.17 and 88.80%, respectively, of the total variance in bacteria and fungi in the vegetation restoration types (Figures 3A,B). By contrast, at the genus level, the first two axes (PC1 and PC2) explained 56.01 and 50.82%, respectively, of the total variation in bacteria and fungi (Figures 3C,D).

Tests of intergroup differences using ANOSIM similarity analysis (number of permutations = 999) showed that, at the phylum level, no significant difference was found in the composition of soil bacterial communities (*p* = 0.108) (Figure 3A), but significant differences were observed in the composition of fungal communities (*p* = 0.001) among the five patterns (Figure 3B). On the contrary, at the genus level, a significant difference was found in the composition of soil bacterial communities (*p* = 0.001) and soil fungal communities (*p* = 0.001) (Figures 3C,D).

### 3.6 Correlation analysis between soil microbial communities and soil environmental factors

**Figure 4:**
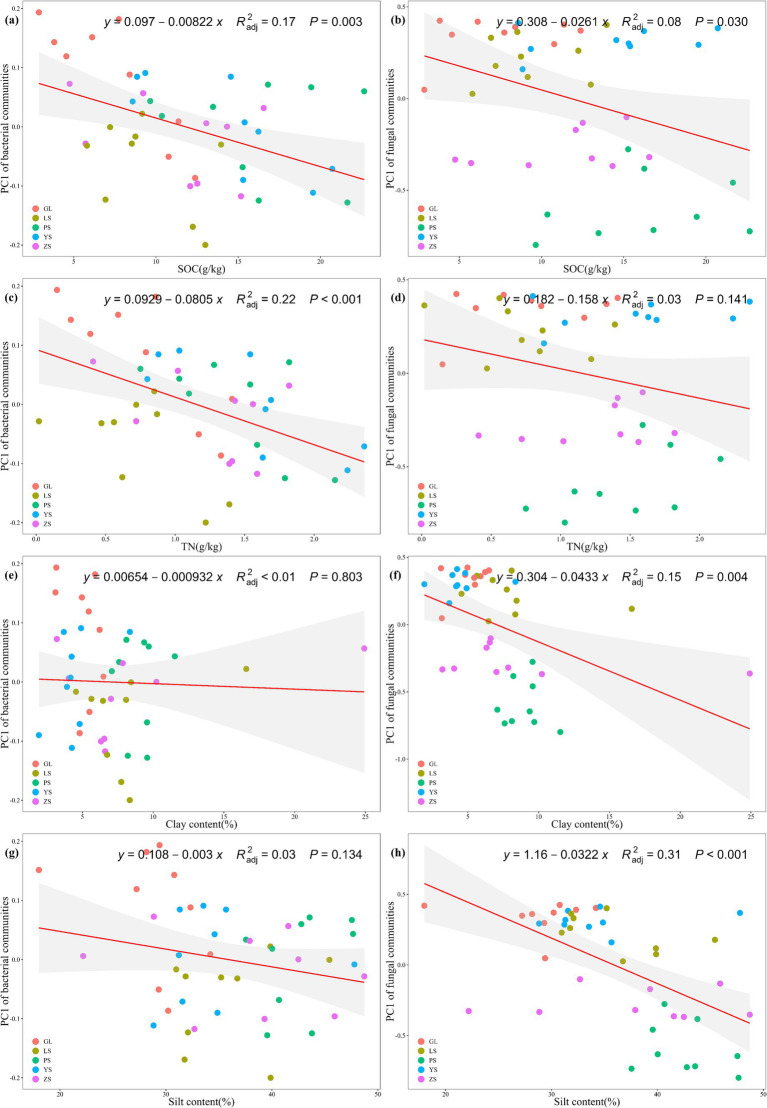
Regression analysis reveals the relationship between microbial community β-diversity on genus level and soil parameters. **(A)** Correlation between soil organic carbon (SOC) and bacterial community β-diversity; **(B)** correlation between soil organic carbon (SOC) and fungal community β-diversity; **(C)** correlation between total nitrogen (TN) and bacterial community β-diversity; **(D)** correlation between total nitrogen (TN) and fungal community β-diversity; **(E)** correlation between clay and bacterial community β-diversity; **(F)** correlation between clay and fungal community β-diversity; **(G)** correlation between silt and bacterial community β-diversity; **(H)** correlation between silt and fungal community β-diversity. GL, nature grassland; LS, larch; PS, populus; YS, ulmus; ZS, pinus.

In this study, after removing redundant variables, four environmental parameters were selected. The results demonstrated that the microbial community structure was driven by various soil environmental factors. Specifically, SOC (*p* = 0.003) and TN (*p* < 0.001) significantly influenced the bacterial community *β*-diversity (Figures 4A,C), while SOC (*p* = 0.030), clay content (*p* = 0.004) and silt content (*p* < 0.001) significantly influenced fungal community β-diversity (Figures 4B,F,H). TN did not affect the fungal community β-diversity (*p* > 0.05) (Figure 4D). Clay content and silt content did not affect the bacterial community β-diversity (both *p* > 0.05) (Figures 4E,G). Bacterial and fungal community structure was influenced by different environmental factors.

### 3.7 Soil microbial community co-occurrence network analysis

**Figure 5:**
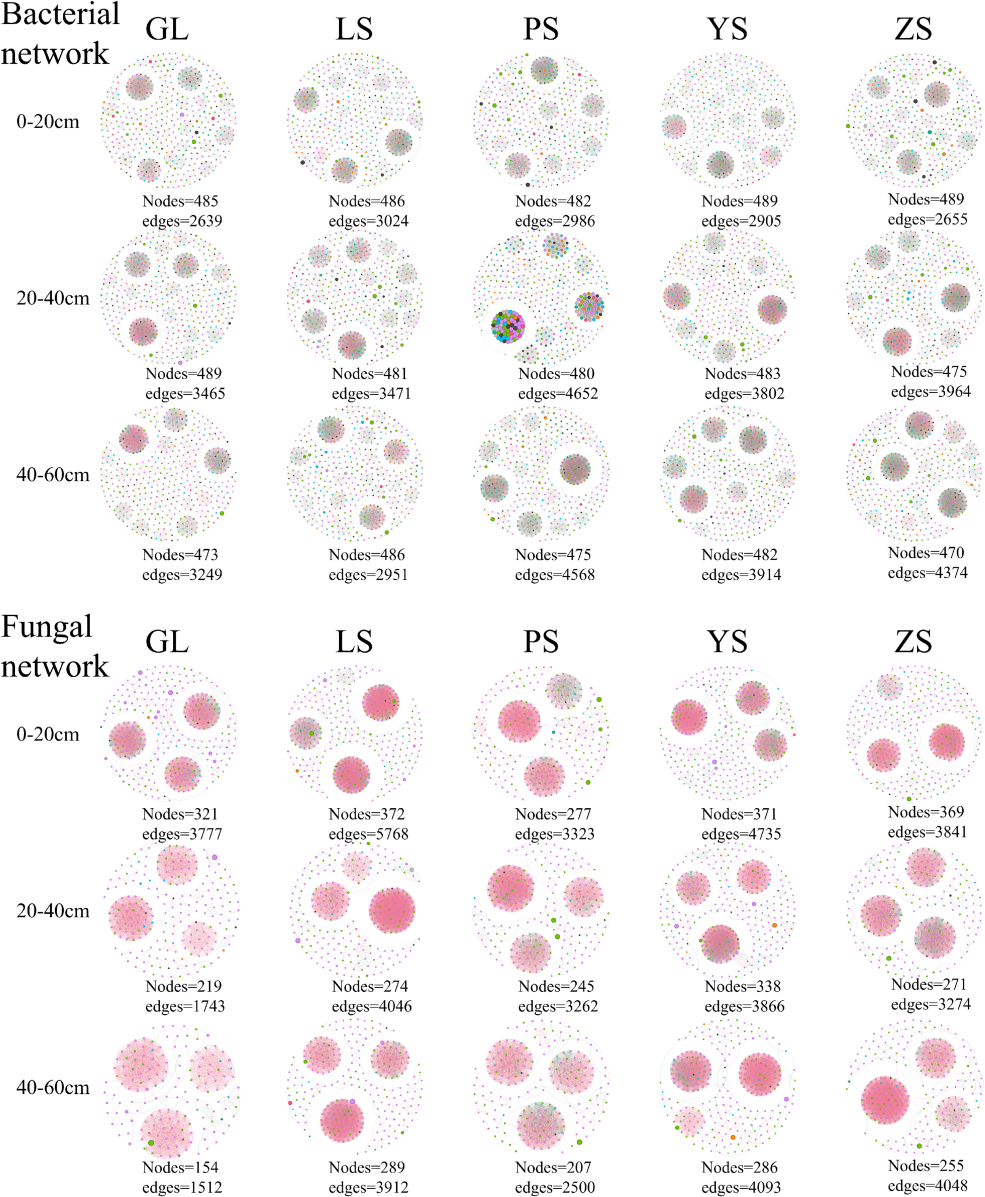
Microbial co-occurrence networks in five different vegetation restoration types at genus level. Each species are represented by nodes, and edges show Pearson’s correlation (*p* > 0.08) and significance values (*p* < 0.01). Co-presence relationships (or positive edges) are colored red, while mutual exclusion relationships (or negative edges) are green. GL, nature grassland; LS, larch; PS, populus; YS, ulmus; ZS, pinus.

The results of the analyses showed that soil bacterial and fungal community networks in various soil layers of different vegetation types exhibited different co-occurrence patterns (Figure 5). Among the five vegetation restoration types, the bacterial and fungal network complexity indices of GL were the lowest, with the sum total of each soil layer being −0.601 and −1.425, respectively (Tables 3, 4). The bacterial network complexity index of PS was the highest (1.108) and the fungal network complexity index of LS was the highest (0.802). Broadleaf forests (PS, YS) having higher bacterial network complexity than coniferous forests (ZS, LS), and coniferous forests having higher bacterial network complexity than natural grassland (GL). This trend is similar to that in soil carbon and nitrogen.

**Table 3:** Parameters of the molecular ecological network of soil bacteria.

Vegetation type	Soil depth/cm	Positive edges (%)	Negative edges (%)	Average degree	Modularity	Density	Average path length	Average clustering coefficient	Complexity index
GL	0–20	55.70	44.30	10.882	0.86	0.022	2.473	0.877	−0.514
	20–40	64.39	35.61	14.172	0.811	0.029	1.348	0.921	0.207
	40–60	65.07	34.93	13.738	0.808	0.029	1.369	0.905	−0.293
	Sum	185.16	114.84	38.792	2.479	0.080	5.190	2.703	−0.601
LS	0–20	52.65	47.35	12.444	0.838	0.026	1.480	0.902	−0.190
	20–40	55.20	44.80	14.432	0.843	0.030	1.224	0.931	0.116
	40–60	61.23	38.77	12.144	0.851	0.025	3.041	0.864	−0.263
	Sum	169.08	130.92	39.020	2.532	0.081	5.745	2.697	−0.338
PS	0–20	53.85	46.15	12.390	0.843	0.026	1.500	0.900	−0.303
	20–40	59.09	40.91	19.383	0.708	0.040	1.179	0.919	0.777
	40–60	60.22	39.78	19.234	0.743	0.041	1.534	0.933	0.635
	Sum	173.16	126.84	51.007	2.294	0.107	4.213	2.752	1.108
YS	0–20	55.15	44.85	11.881	0.854	0.024	2.532	0.854	−0.340
	20–40	65.28	34.72	15.743	0.794	0.033	2.237	0.905	0.232
	40–60	56.41	43.59	16.241	0.819	0.034	1.129	0.912	0.377
	Sum	176.84	123.16	43.865	2.467	0.091	5.898	2.671	0.269
ZS	0–20	51.79	48.21	10.859	0.864	0.022	2.033	0.919	−0.317
	20–40	56.86	43.14	16.691	0.804	0.035	1.326	0.804	−0.143
	40–60	52.58	47.42	18.613	0.792	0.040	1.198	0.792	0.022
	Sum	161.23	138.77	46.163	2.460	0.097	4.557	2.515	−0.438

GL, nature grassland; LS, larch; PS, populus; YS, ulmus; ZS, pinus.

**Table 4:** Parameters of the molecular ecological network of soil fungal.

Vegetation type	Soil depth/cm	Positive edges (%)	Negative edges (%)	Average degree	Modularity	Density	Average path length	Average clustering coefficient	Complexity index
GL	0–20	82.42	17.58	23.101	0.697	0.071	1.483	0.880	−0.354
	20–40	97.48	2.52	15.918	0.644	0.073	1.087	0.921	−0.778
	40–60	98.74	1.26	19.636	0.665	0.128	1.164	0.957	−0.293
	Sum	278.64	21.36	58.655	2.006	0.272	3.734	2.758	−1.425
LS	0–20	83.67	16.33	31.011	0.650	0.084	1.122	0.912	0.603
	20–40	96.89	3.11	29.425	0.464	0.107	1.263	0.889	0.082
	40–60	93.05	6.95	27.647	0.611	0.098	1.057	0.902	0.117
	Sum	273.61	26.39	88.083	1.725	0.289	3.442	2.703	0.802
PS	0–20	89.50	10.50	23.993	0.662	0.087	2.060	0.949	0.127
	20–40	89.42	10.58	26.520	0.617	0.108	1.039	0.950	0.248
	40–60	86.64	13.36	24.155	0.684	0.117	1.106	0.949	0.007
	Sum	265.56	34.44	74.668	1.963	0.312	4.205	2.848	0.383
YS	0–20	88.22	11.78	25.526	0.684	0.069	1.225	0.892	0.041
	20–40	89.03	10.97	22.876	0.648	0.068	1.268	0.900	−0.203
	40–60	92.82	7.18	28.622	0.586	0.100	1.112	0.901	0.151
	Sum	270.07	29.93	77.024	1.918	0.237	3.605	2.693	−0.011
ZS	0–20	89.61	10.39	20.818	0.626	0.057	1.245	0.917	−0.173
	20–40	86.32	13.68	24.162	0.689	0.089	1.069	0.925	−0.052
	40–60	95.01	4.99	31.749	0.503	0.125	1.052	0.920	0.476
	Sum	270.94	29.06	76.729	1.818	0.271	3.366	2.762	0.251

GL, nature grassland; LS, larch; PS, populus; YS, ulmus; ZS, pinus.

## 4 Discussion

### 4.1 Effects of vegetation restoration types on soil properties

In this study, compared with natural grassland, after about 50 years of vegetation restoration, four plantation forests had a increase of soil nutrients (TC, TN, SOC). Especially, populus had significantly higher TC, TN and SOC than natural grassland in each soil layer (*p* < 0.05). From the view of improving soil quality (carbon and nitrogen), populus are the better choices of tree species for plantation forest restoration types in the agricultural pastoral ecotone of Zhangjiakou. Populus are considered the king of the world’s fast-growing tree species, with a rapid growth rate and large annual increase (Łukaszkiewicz et al., 2024). It is able to absorb and store large amounts of carbon dioxide in a relatively short period of time (Bahrian et al., 2024; Mulyana et al., 2024). In addition, populus have a symbiotic relationship with certain nitrogen-fixing bacteria that can help them fix more nitrogen, thereby increasing the nitrogen content of the soil and improving its fertility. This study revealed that broadleaf forests (PS, YS) had higher TC than coniferous forests (ZS, LS), and coniferous forests had higher TC than natural grassland (GL). This trend was attributed to the fact that broadleaf had a thicker layer of litter that usually decomposes faster than that in coniferous forests, which, in turn, increases the carbon input to the soil (Wei et al., 2021). Previous studies have revealed higher soil carbon accumulation in broadleaf forests than in coniferous forests in northern area, which is similar to the results of our study (Geng et al., 2009; Wei et al., 2021). Particle size analysis revealed that the soil in the study area had a higher content of sand particles and a lower content of silt and clay particles, which are more susceptible to erosion and transport by monsoon winds due to their smaller size and lighter mass (Chen et al., 2020). Compared to grassland, plantation forests play a role in reducing wind speed and slowing down wind erosion. Thus, fine particles carried by the atmosphere are intercepted, which leads to the gradual accumulation of more fine materials in the soil surface layer (Guo et al., 2020). This phenomenon is reflected in the higher content of clay and powder particles in plantation forests.

### 4.2 Effect of vegetation restoration types on soil microbial community structure

Our results showed that bacterial α-diversity was significantly higher in ZS and LS than that in GL in the 20–40 cm soil layer. Plantation forests have a large and complex root systems, which provides a comfortable habitat for soil microbial communities. This condition promotes soil microbial massive uptake of soil nutrients and have an increase of microbial α-diversity (Yang et al., 2020b). However, fungal α-diversity was significantly lower in PS than that in GL in the 0–20 cm soil layer. The plantation forest vegetation in this study was all monospecific, with low litter diversity in the top soil layer (0–20 cm), which also had a negative impact on microbial α-diversity (Otsing et al., 2018; Xu et al., 2022). Regression analyses showed that SOC and TN were significantly correlated with soil bacterial community composition; SOC, clay content and silt content were significantly correlated with soil fungal community composition. This study revealed that SOC were both significantly correlated with soil bacterial and fungal community composition. Since SOC serves as the primary source of energy and nutrients for soil microorganisms, and different microorganisms have different efficiencies and preferences in utilizing SOC, changes in SOC can significantly influence soil microbial community composition (Sokol et al., 2022). Furthermore, these findings implied that the bacterial community composition was regulated by soil nutrients to a certain extent in the process of vegetation restoration in agricultural pastoral ecotone, while the fungal community structure is mainly regulated by soil texture. This result was similar to findings by Xia et al. that soil texture has a greater effect on fungi than bacteria (Xia et al., 2020).

### 4.3 Effect of vegetation restoration types on microbial co-occurrence network

Our results showed that the microbial network complexity increased with soil nutrients. Overall, the microbial network complexity of GL was lowest in five vegetation types, which is similar to the findings of Yang et al. (2020a, 2020b). A rise in carbon and nitrogen content can increase the number of soil microorganisms, which will lead to the formation of a more complex soil microbial network (Zhou et al., 2020; Jiao et al., 2021). In this study, the complexity of soil microbial network in plantation forest vegetation gradually increased with the improvement in soil nutrient status. The restoration of plantation forest vegetation enhanced microbial community stability, which is important for slowing down soil wind erosion, resisting environmental disturbances, and promoting the cycling of soil nutrients (Wang et al., 2023). Especially, the bacterial network complexity of PS was highest in five vegetation types. We mentioned above that populus are the better choices of tree species to improve soil quality. Now we do believe that populus are very suitable for planting in the agricultural pastoral ecotone of Zhangjiakou. Complex microbial networks not only enhance plant growth and resilience, but also improve ecosystem stability and sustainability and promote the long-term benefits of environmental restoration (Jagadesh et al., 2024).

We also found that the modularity index and average path length were higher for bacteria than for fungi. The network modularity index usually characterizes the resistance of the system to external disturbances, with higher modularity indexes being associated with greater resistance to external disturbances (Deng et al., 2012). A smaller average path length indicates a closer interaction among network nodes, which also implies a faster diffusion of external disturbances in the microbial network and a more sensitive response to disturbances (Carpenter et al., 2012). This observation suggests that bacterial networks are more stable than fungal networks in responding to disturbances, which is consistent with previous studies (Zhao et al., 2019). Bacteria are able to cope with drought through rapid reproduction, dormancy and metabolic adjustments (Lebre et al., 2017), while fungi are more dependent on water and moist environments, rendering them susceptible to greater impacts under drought conditions (Logan et al., 2021). After drought or other disturbances, bacteria are able to restore ecological functions more quickly and promote ecosystem restoration. In contrast, fungi are slower to recover and may slow down the ecosystem restoration process. In conclusion, more attention should be paid to bacterial network characteristics during dryland vegetation restoration.

## 5 Conclusion

Overall, this study highlights the importance of soil quality, microbial diversity and network complexity in plantation forest and natural grassland in Zhangjiakou Tunken Forest Farm. There is a fact that soil TC content is highest in broadleaf forests, followed by coniferous forests and natural grassland. In addition, we have shown that soil bacteria and fungi had different drivers, and soil organic carbon is a key driver regulating both bacterial and fungal community composition. Compared to natural grassland, soil microbial networks became more complex in plantation forests restoration types (*P. sylvestris* var. *mongholica*, *L. principis- rupprechtii*, *P. tomentosa*, and *U. pumila*), especially the highest bacterial network complexity in *P. tomentosa* soil. Anyway, the improvement of both soil quality and microbial network complexity should be considered when rehabilitating land desertification. Thus, maintaining high levels of soil quality and microbial network complexity sustains the function of ecosystem services in the agricultural pastoral ecotone of China. This will further be greatly beneficial for prevention and control of desertification around the world.

## Data Availability

The original contributions presented in the study are included in the article/supplementary material, further inquiries can be directed to the corresponding author.
